# Counting the cost of overfishing on sharks and rays

**DOI:** 10.7554/eLife.02199

**Published:** 2014-02-05

**Authors:** Darcy Bradley, Steven D Gaines

**Affiliations:** 1 **Darcy Bradley** is at the Bren School of Environmental Science and Management, University of California, Santa Barbara, Santa Barbara, United Statesdbradley@bren.ucsb.edu; 2 **Steven D Gaines** is at the Bren School of Environmental Science and Management, University of California, Santa Barbara, Santa Barbara, United Statesgaines@bren.ucsb.edu

**Keywords:** population biology, extinction risk, biodiversity change, fishes, sharks and rays, life histories, other

## Abstract

Over half of all shark and ray species are at risk of extinction or at least heading that way.

**Related research article** Dulvy NK, Fowler SL, Musick JA, Cavanagh RD, Kyne PM, Harrison LR, Carlson JK, Davidson LNK, Fordham SV, Francis MP, Pollock CM, Simpfendorfer CA, Burgess GH, Carpenter KE, Compagno LJV, Ebert DA, Gibson C, Heupel MR, Livingstone SR, Sanciangco JC, Stevens JD, Valenti S, White WT. 2014. Extinction risk and conservation of the world’s sharks and rays. *eLife* **3**:e00590. doi: 10.7554/eLife.00590

**Image** The thresher shark is threated with extinction in much of its range (NMFS/PRIO Observer Program)

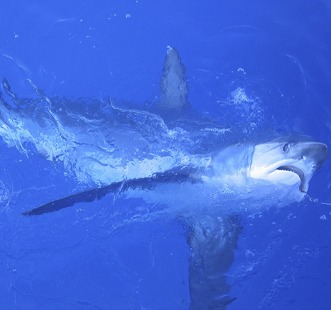

Chondrichthyans—the class of fish that includes sharks and rays—are in a bad, bad way. Their numbers have plummeted, mostly due to overfishing, which is largely driven by the demand for shark fin soup (Jackson et al., 2001; Myers and Worm, 2003). All attempts at saving species have fallen short, not because of a lack of concern, but instead because of a lack of data. It is difficult to know just how depleted sharks and rays are in number, just as it is difficult to determine how and where conservation efforts are most desperately needed. This is concerning not just for chondrichthyans, but also for entire ecosystems, because the removal of large-bodied predators, such as sharks, can cause entire food webs to collapse (Stevens et al., 2000; Mumby et al., 2006; Heithaus et al., 2008).

To address this knowledge gap, Nicholas Dulvy of Simon Fraser University and co-workers in Canada, UK, USA, Australia, New Zealand and South Africa have performed a systematic evaluation of the relative extinction risk for more than 1000 species of sharks, rays and the less well known chimaeras (Dulvy et al., 2014). Their findings—which have been published in *eLife*—are alarming, but more importantly, the story they reveal helps to frame the chondrichthyan problem in ways that can help guide effective solutions.

Overfishing can be a threat anywhere, to any species, yet sharks and rays share characteristics that make them particularly vulnerable. They mature late, they have a long gestation period, and they create few offspring. Moreover, they have large ranges, often spanning waters belonging to more than one nation, so efforts to protect them require international coordination. Furthermore, as they are overfished and their populations drop, the commercial value of these fish only increases, incentivizing further overharvesting.

Dulvy et al. expose a staggering result: more than half of all chondrichthyan species are predicted to be ‘Threatened or Near Threatened’ according to the Red List maintained by the International Union for the Conservation of Nature. By comparison, insects, mammals and amphibians are all under less threat (Figure 1).

**Figure 1.**
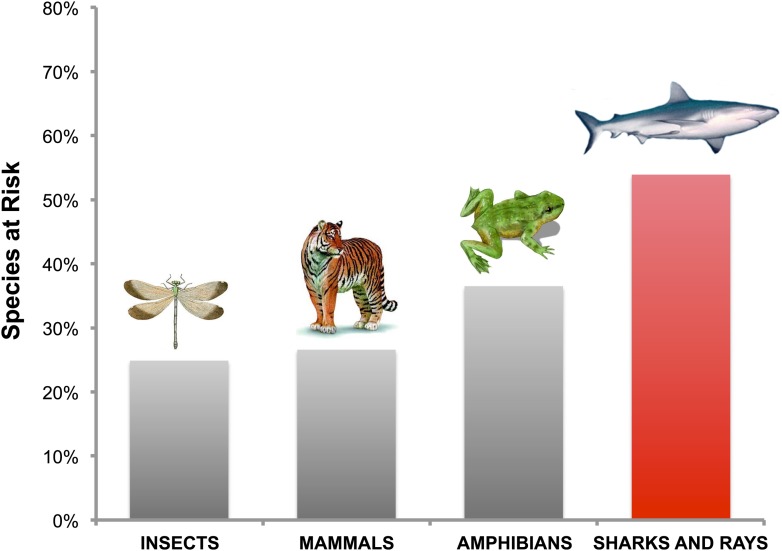
Sharks and rays are more under threat than insects, mammals and amphibians. According to the Red List of Threatened Species maintained by the International Union for the Conservation of Nature (IUCN), 1148 species of insects (24.9% of the total), 1467 species of mammals (26.6%) and 2339 species of amphibians (36.5%) are ‘Threatened or Near Threatened’. Dulvy et al. estimate that for sharks and rays this figure is 562 species (53.9% of the total). The IUCN definition of Threatened includes species that are Critically Endangered, Endangered or Vulnerable.

Illustrations: Dru Drury, USDA APHIS, and Nobu Tamura. Photograph: Chris Huh.

One of the biggest challenges to compiling such estimates of global threat is that there are very limited data available for many species. Indeed, nearly half of the shark and ray species are formally classified as ‘Data Deficient’, which is one of the highest proportions of any class of species (Hoffmann et al., 2010). To overcome this challenge, which is common for all species at risk, Dulvy et al. used information about those species of sharks and rays for which abundant data were available to derive general patterns that are associated with a higher risk of extinction. By classifying the attributes of these different species—by answering questions such as, where do they live, how deep do they swim, what size are they—Dulvy et al. were able to generate model predictions for the likely status of species with more limited data.

They found that the most useful factors for determining if a particular species had an elevated risk of extinction was its maximum body size, the minimum depth of water in which it lived, and the range of depth—with larger species and those that swim in shallower waters having the largest risk. Although geographic range is closely linked to extinction risk in many groups of animals, it is largely unrelated to the extinction risk of sharks and rays. These threat patterns highlight the devastating impact of fishing on chondrichthyans—shark and ray fishing activity is now so ubiquitous that only species with broad depth ranges can escape from fishing gear.

Forecasting the extinction risk of sharks and rays can guide future management actions and policy decisions—especially for those species without sufficient data to allow more formal assessments of their status. For example, the enormous variation between regions in the status of sharks and rays evident in the findings of Dulvy et al. provides scope for setting region specific conservation priorities. It should also allow us to identify examples of current successes—where shark and ray populations are doing well—that we will need to replicate to secure the long-term future survival of these fish.

In addition, an important pattern that has emerged in global analyses of other fished species is that fisheries with more definite estimates of their stock status tend to be in substantially better condition than fisheries with limited information (Worm et al., 2009; Costello et al., 2012). This information is also valuable for conservation efforts, as it is hard to make effective decisions in the absence of fact. Although the estimates of species status in this new study still have large uncertainties, they do provide an important step towards gaining information that can drive more effective conservation and management decisions.

As we look to the future of sharks and rays, one key challenge lies in first developing species assessments with better estimates of the populations involved. These assessments can then be linked with effective management practices that have been successfully employed in large numbers of global fisheries. Dulvy et al. stress that it is unclear whether the declining populations of sharks and rays that live around the world can be reversed on a local scale. Instead, these trends could be symptomatic of some long-term and widespread accumulation of extinction risk across the world’s seas and oceans. The insight from this new global analysis enhances the chance for recovery if these findings help drive effective local and collaborative action.
